# Disc haemorrhage associated with an enlarged peripapillary intrachoroidal cavitation in a non-glaucomatous myopic eye: a case report

**DOI:** 10.1186/s12886-015-0143-7

**Published:** 2015-10-29

**Authors:** Kyoung Min Lee, Eun Ji Lee, Seung Hyen Lee, Tae-Woo Kim

**Affiliations:** Department of Ophthalmology, Seoul National University Bundang Hospital, Gyeonggi-do, Seongnam-si, Bundang-gu Republic of Korea

**Keywords:** Disc haemorrhage, Peripapillary intrachoroidal cavitation, Optical coherence tomography, Myopia

## Abstract

**Background:**

Disc haemorrhage (DH) is considered a characteristic sign of glaucoma, but its causative mechanism remains to be determined. We present a case of DH that occurred in association with an enlarged peripapillary intrachoroidal cavitation in a non-glaucomatous eye.

**Case presentation:**

A 35-year-old woman was evaluated for a DH that had been detected during a preoperative examination for myopic refractive surgery. Enhanced depth imaging spectral-domain optical coherence tomography imaging of the optic nerve revealed a peripapillary intrachoroidal cavitation adjacent to the DH. The DH was also present at the 1-year follow-up, but had been completely absorbed at the 2-year follow-up, respectively with an enlargement and shrinkage of the intrachoroidal cavitation and prelaminar tissue schisis. Glaucomatous optic nerve change was not observed during the entire follow-up.

**Conclusion:**

DH can be caused by mechanical damage to capillaries from microscopic changes in peripapillary tissues such as enlargement of the intrachoroidal cavitation, regardless of the presence of glaucoma.

**Electronic supplementary material:**

The online version of this article (doi:10.1186/s12886-015-0143-7) contains supplementary material, which is available to authorized users.

## Background

Disc haemorrhage (DH) is a characteristic of glaucomatous optic nerve damage, but it is reported to also occur in eyes without glaucoma [1–5]. The mechanism and clinical significance of DH in healthy subjects is not fully understood. Here we describe a case of DH in a non-glaucomatous eye that was associated with an enlarged peripapillary intrachoroidal cavitation (PIC) [6, 7].

## Case presentation

**Fig. 1:**
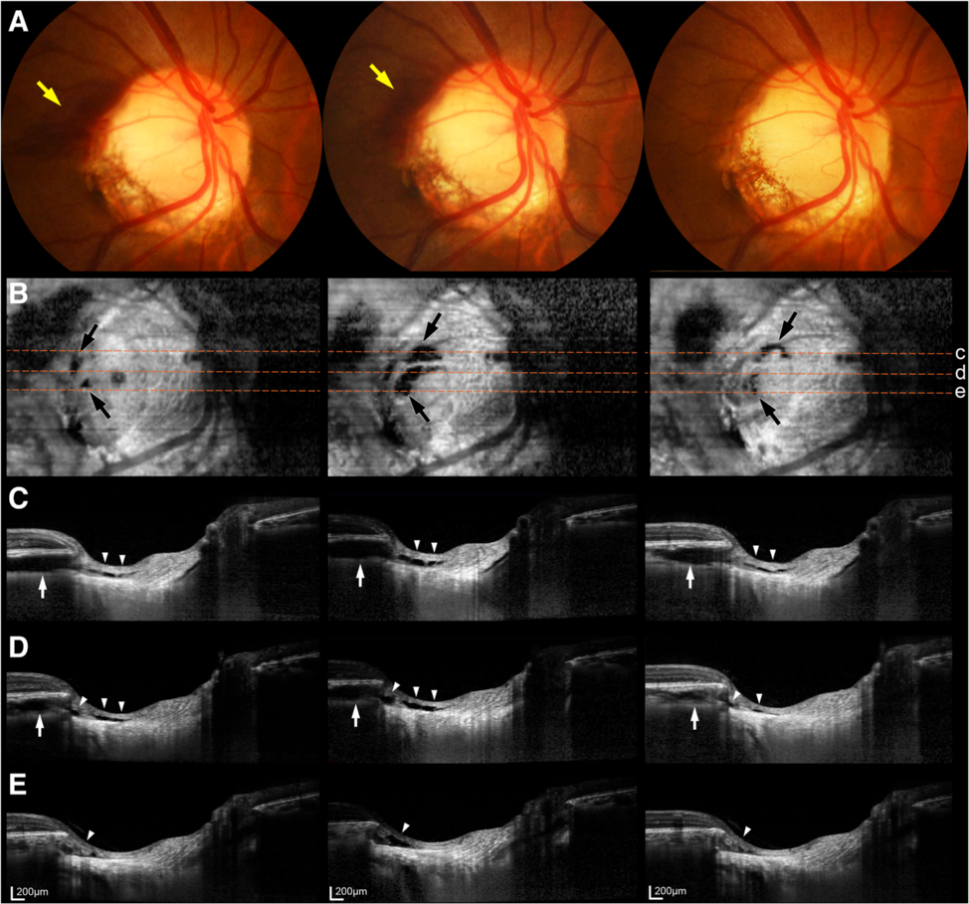
Colour disc photographs (**a**), en-face spectral-domain optical coherence tomography images at the level of the peripapillary choroidal cavitation and prelaminar tissue schisis (**b**), and B-scan images at the location indicated by the orange dashed lines (**c**–**e**) obtained at the first visit (*left*) and at the follow-up visits after 1 year (*middle*) and 2 years (right). **a** A flame-shaped haemorrhage was observed at the first two visits (*yellow arrows*), which was absorbed at the final follow-up. **b** En-face images showed the enlarged fluid pockets within the prelaminar tissue at the second visit (middle, *black arrows*), which again were smaller at the final visit (right, *black arrows*). **c**–**e** Both the intrachoroidal cavitation (*white arrows*) and prelaminar tissue schisis (*arrowheads*) were larger at the second visit (*middle*) than at the first visit (*left*), and then smaller at the final visit

This case report adheres to CARE guidelines. A previously healthy female aged 35 years was referred to our hospital for evaluation of a DH in her right eye that had been detected during a preoperative examination for myopic refractive surgery. At her first visit, the patient’s best corrected visual acuity was 20/20 OU, the refractive error was −4.75 diopters OD and −5.75 diopters OS, and the intraocular pressure was 13 mmHg OU. The anterior segment was unremarkable bilaterally, while a dilated funduscopic examination of the right eye revealed a diffuse flame-shaped haemorrhage at the superotemporal optic disc border (Fig. 1a, left). Enhanced depth imaging (EDI) spectral-domain optical coherence tomography (SD-OCT) volume scanning of the optic disc revealed an intrachoroidal cavitation in the superotemporal peripapillary area (Fig. 1b–e, left), adjacent to the DH. Localized prelaminar tissue schisis was observed in connection with the PIC (Fig. 1b–e, left). A funduscopic examination and SD-OCT images of the optic disc did not reveal any signs of vitreopapillary traction. Stereo disc photography, red-free fundus photography, and an SD-OCT evaluation of the thickness of the peripapillary retinal nerve fibre layer showed no evidence of glaucomatous optic nerve damage in her right eye.

One year later a DH was observed at the same location, although it was smaller (Fig. 1a, middle). EDI SD-OCT optic disc scanning revealed a slightly enlarged choroidal cavitation, with an apparently enlarged schisis of the prelaminar tissue (Fig. 1b–e, middle). Two years later the DH was completely absorbed (Fig. 1a, right), and both the choroidal cavitation and prelaminar tissue schisis were smaller (Fig. 1b–e, right; Additional file 1: Video S1). There was no change in either the visual acuity or refractive error of the right eye. The intraocular pressure was 11 mmHg OD and the findings of a glaucoma evaluation were still unremarkable.

## Discussion

The pathogenesis of DH remains unclear. The mechanical hypothesis holds that DH in glaucoma is caused by microvascular disruption from stretching of capillaries due to structural changes in the optic nerve head tissues [8, 9] or atrophic changes in the retinal nerve fibre layer with glaucoma progression [10].

In our case, the DH was associated with PIC and was observed in a non-glaucomatous myopic eye. It is unclear whether the DH was caused by the enlarged PIC or prelaminar tissue schisis, or whether the presence of these two features was coincidental. However, we speculate that the occurrence of DH was associated with the enlarged PIC and prelaminar tissue schisis, because these features that presented with the DH were smaller after DH absorption. Although the time required for a DH to be absorbed may vary according to its size, location and cause, it is generally acknowledged that a DH disappears within several months [11]. Our case exhibited DHs at both visits within a 1-year interval, which means that it is possible that the DH recurred during that interval, with the DHs evident at the two visits occurring during the course of PIC and prelaminar tissue schisis formation or enlargement. We hypothesize that the extra fluid within the PIC entered the prelaminar tissue and aggravated the prelaminar schisis so as to stretch and damage the adjacent peripapillary vessels [12, 13]. On the other hand, it is also possible that the DH in our case was caused by retinal vessels that were in an abnormal condition. An angiographic examination might have been useful in more clearly ruling out a retinal vascular pathology for the cause of DH.

The pathogenesis and clinical importance of PIC are poorly understood, and the relationship between PIC and glaucoma is unclear [14]. It is therefore uncertain whether DH associated with PIC indicates an increased susceptibility to glaucomatous optic nerve damage. Although a longer term follow-up is required, the glaucomatous damage was unremarkable during the 2 years of follow-up in the present case.

## Conclusions

DH can appear as a manifestation of peripapillary structural change. The mechanical damage to peripapillary capillaries associated with PIC enlargement may be one of the causes of DH in eyes without glaucoma.

## Consent

Written informed consent was obtained from the patient for publication of this case report and any accompanying images. A copy of the written consent is available for review by the Editor of this journal.

## Additional file

Additional file 1:
**Video S1.** Swept-source optical coherence tomography images of the optic nerve head obtained at the second (upper) and final (lower) visits. The images were obtained using the DRI-OCT1 system (Atlantis, Topcon, Tokyo, Japan) in a three-dimensional raster-scan protocol acquiring 256 × 256 A-scans per data. Note the decreased size of both the intrachoroidal cavitation and prelaminar tissue schisis at the final visit (lower) compared to the second visit (upper). (MPEG 1667 kb)

## Abbreviations

DH: disc hemorrhage
EDI: enhanced depth imaging
PIC: peripapillary intrachoroidal cavitation
SD-OCT: spectral-domain optical coherence tomography
